# Polyamine Oxidase Expression Is Downregulated by 17β-Estradiol via Estrogen Receptor 2 in Human MCF-7 Breast Cancer Cells

**DOI:** 10.3390/ijms23147521

**Published:** 2022-07-07

**Authors:** Jin Hyung Kim, Seung-Taek Lee

**Affiliations:** Department of Biochemistry, College of Life Science and Biotechnology, Yonsei University, Seoul 03722, Korea; qnt1313@yonsei.ac.kr

**Keywords:** 17β-estradiol (E2), polyamine, polyamine oxidase (PAOX), estrogen receptor (ESR), activator protein 1 (AP-1)

## Abstract

Polyamine levels decrease with menopause; however, little is known about the mechanisms regulated by menopause. In this study, we found that among the genes involved in the polyamine pathway, polyamine oxidase (*PAOX*) mRNA levels were the most significantly reduced by treatment with 17β-estradiol in estrogen receptor (ESR)-positive MCF-7 breast cancer cells. Treatment with 17β-estradiol also reduced the PAOX protein levels. Treatment with selective ESR antagonists and knockdown of ESR members revealed that estrogen receptor 2 (ESR2; also known as ERβ) was responsible for the repression of PAOX by 17β-estradiol. A luciferase reporter assay showed that 17β-estradiol downregulates *PAOX* promoter activity and that 17β-estradiol-dependent PAOX repression disappeared after deletions (−3126/−2730 and −1271/−1099 regions) or mutations of activator protein 1 (AP-1) binding sites in the *PAOX* promoter. Chromatin immunoprecipitation analysis showed that ESR2 interacts with AP-1 bound to each of the two AP-1 binding sites. These results demonstrate that 17β-estradiol represses *PAOX* transcription by the interaction of ESR2 with AP-1 bound to the *PAOX* promoter. This suggests that estrogen deficiency may upregulate PAOX expression and decrease polyamine levels.

## 1. Introduction

Polyamines are polycationic alkylamines and include spermidine, spermine, and the precursor diamine putrescine. These small water-soluble molecules are fully protonated in cells owing to the presence of primary and secondary amino groups. They can easily bind to a range of negatively charged molecules, including RNA, DNA, and certain types of proteins and phospholipids. Thus, polyamines are involved in gene regulation, protein and nucleic acid synthesis, and DNA stability and play a role in many physiological processes, including cell proliferation and differentiation [1,2,3].

Polyamines are tightly controlled and regulated by enzymes involved in the anabolism and catabolism of polyamines [4,5]. Ornithine decarboxylase 1 (ODC1) catalyzes the first rate-limiting step of polyamine biosynthesis, which converts ornithine to putrescine. S-adenosylmethionine (SAM) decarboxylase 1 (AMD1) catalyzes the conversion of SAM into decarboxylated SAM. Putrescine promotes the conversion of the AMD1 proenzyme into the active enzyme and enhances AMD1 activity by binding to the allosteric site of AMD1. Spermidine synthase (SRM) converts putrescine into spermidine, and spermine synthase (SMS) converts spermidine into spermine. Spermidine/spermine N1-acetyltransferase 1 (SAT1), polyamine oxidase (PAOX), and spermine oxidase (SMOX) are major enzymes that catalyze the backward conversion of polyamines. SAT1 is a rate-limiting enzyme of polyamine catabolism that catalyzes the N1-acetylation of spermidine or spermine. PAOX is a flavoenzyme that catalyzes the oxidative deamination of N1-acetyl-spermine or N1-acetyl-spermidine to produce spermidine or putrescine. Spermine oxidase (SMOX) is also a flavoenzyme that produces spermidine by the direct oxidation of spermine. The intracellular levels of polyamines are affected by absorption, excretion, biosynthesis, and degradation.

Several factors modulate the regulation of polyamine levels. Certain environmental conditions such as drought, starvation, and heat and cold shock, which trigger a polyamine-stress response, increase polyamine production [6]. In contrast, smoking [7] and alcohol consumption [8] reduce polyamine synthesis by decreasing stress. Polyamine levels are known to be high in several cancers, such as breast, lung, liver, ovarian, colon, and pancreatic cancers [4]. In contrast, kidney diseases such as chronic renal failure, diabetic nephropathy, and chronic glomerulonephritis are characterized by low polyamine levels [9]. In addition, aging gradually decreases polyamine levels in various organs [10,11,12]. Spermidine levels decrease from adulthood to old age in several regions of the brain, including the basal ganglia [12].

Menopause is characterized by a decrease in the secretion of the ovarian hormone estrogen as the ovaries are depleted and the permanent cessation of ovarian follicular activity [13,14]. Estrogen deficiency due to menopause has diverse implications, including a decline in dermal collagen content [15], cardiovascular deterioration [16], hair loss [17], nervous system imbalances [18], and loss of bone density [19,20].

Estrogen is known to affect polyamine metabolism. It has also been reported that polyamine levels decrease during menopause [21]. Estrogen stimulation increases the synthesis and accumulation of polyamines in ovariectomized rats [22]. The reduction in polyamines is responsible for various diseases, such as hair loss [23,24], ischemic brain [25], neurodegenerative diseases [26], collagen-induced arthritis [27], and osteoporosis [28]. Some disorders associated with polyamine deficiency overlap with the clinical implications of menopause.

Previous studies have shown a clear association between estrogen deficiency and reduced polyamines [29,30]. However, the molecular mechanism by which decreased estrogen levels lower polyamine levels has not been elucidated. Therefore, we sought to identify the enzyme via which estrogen deficiency manifests polyamine reduction, as well as to analyze the regulation of the target enzyme by estrogen. To this end, we investigated the effects of 17β-estradiol (E2) on the expression of enzymes involved in the polyamine metabolic pathway in estrogen receptor (ESR)-positive MCF-7 breast cancer cells. We found that *PAOX* expression was most strongly affected by E2. The estrogen receptor responsible for the repression of PAOX expression by E2 was also determined. The E2-responsive regions of the *PAOX* promoter were identified using a luciferase reporter assay. A transcription factor that binds to the E2-responsive regions of the *PAOX* promoter and the interaction of the transcription factor with the E2-estrogen receptor were analyzed using chromatin immunoprecipitation. Based on these results, we propose a molecular mechanism for estrogen-mediated PAOX repression.

## 2. Results

### 2.1. E2 Changes PAOX mRNA Levels Most among the Genes Involved in Polyamine Metabolism

**Figure 1:**
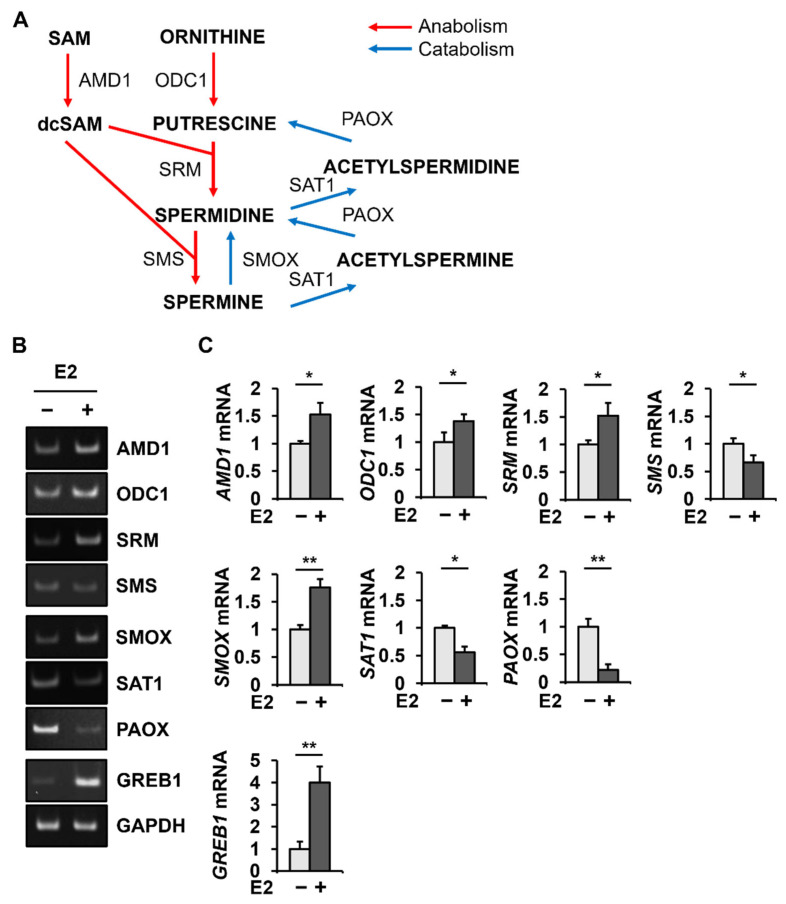
Among the genes involved in the polyamine metabolic pathway, *PAOX* is most significantly altered by E2 treatment at the RNA level. Schematic diagram of the polyamine metabolic pathway (**A**). MCF-7 cells were incubated with or without E2 for 24 h. Levels of *AMD1*, *ODC1*, *SRM*, *SMS*, *SMOX*, *SAT1*, *PAOX*, *GREB1,* and *GAPDH* mRNAs were evaluated by conventional (**B**) and quantitative (**C**) PCR. Data are shown as the mean ± S.D. (*n* = 3), normalized to *GAPDH* expression. * *p* < 0.05; ** *p* < 0.01 versus control.

The anabolic and catabolic enzymes involved in the polyamine metabolic pathway are shown (Figure 1A). To detect changes in polyamine metabolic enzymes due to E2 treatment, the mRNA levels of polyamine metabolic enzymes were analyzed in MCF-7 cells treated with or without E2 by conventional PCR (Figure 1B) and quantitative PCR (Figure 1C). The data from the conventional PCR were consistent with the quantitative PCR data. The quantitative PCR revealed that the level of *GREB1* mRNA, which is known to be induced by E2 [31], increased to 400.1 ± 72.2% in MCF-7 cells. E2 treatment in MCF-7 cells increased the mRNA levels of *AMD1* (154.9 ± 21.6%), *ODC1* (138.0 ± 12.4%), *SRM* (152.6 ± 22.6%), and *SMOX* (176.0 ± 15.1%) but decreased the mRNA levels of *SMS* (66.5 ± 12.7%), *SAT1* (56.1 ± 10.5%), and *PAOX* (22.6 ± 9.4%).

**Figure 2:**
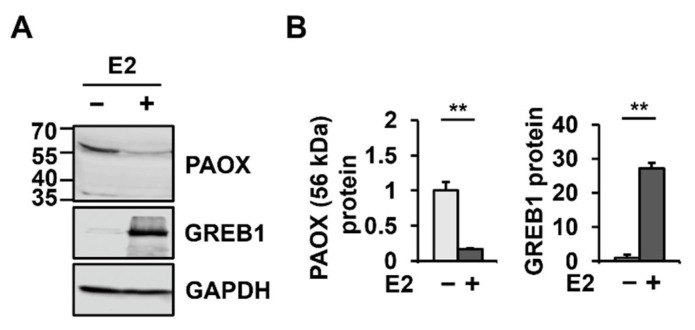
PAOX expression is downregulated at the protein level upon E2 treatment. MCF-7 cells were treated with or without E2 for 24 h. Expression levels of PAOX (56 kDa), GREB1, and GAPDH in cell lysates were evaluated by western blotting using corresponding antibodies (**A**) and were quantitated using the ImageJ software (**B**). Data are shown as the mean ± S.D. (*n* = 3), normalized to *GAPDH* expression. ** *p* < 0.01 versus control.

Among the genes encoding enzymes involved in polyamine metabolism, mRNA levels of *PAOX* showed the most significant change (4.3-fold decrease) after E2 treatment (Figure 1C). Therefore, changes in PAOX expression at the protein level were investigated in MCF-7 cells with or without E2 treatment. As expected, GREB1 levels were significantly increased to 2708.2 ± 164.2% by E2 treatment (Figure 2). PAOX polypeptides were detected as a major band of 56 kDa and a minor band of 35 kDa, suggesting isoform 1 (GenBank NP_690875.1) and isoform 2 (GenBank NP_997010.1), respectively (Figure 2A). Consistent with the E2-dependent decrease in *PAOX* mRNA levels, a significant decrease in levels of both PAOX polypeptides (16.9 ± 1.3% for the major 56-kda band) was observed after E2 treatment (Figure 2B).

### 2.2. E2 Decreases PAOX Expression via the ESR2

**Figure 3:**
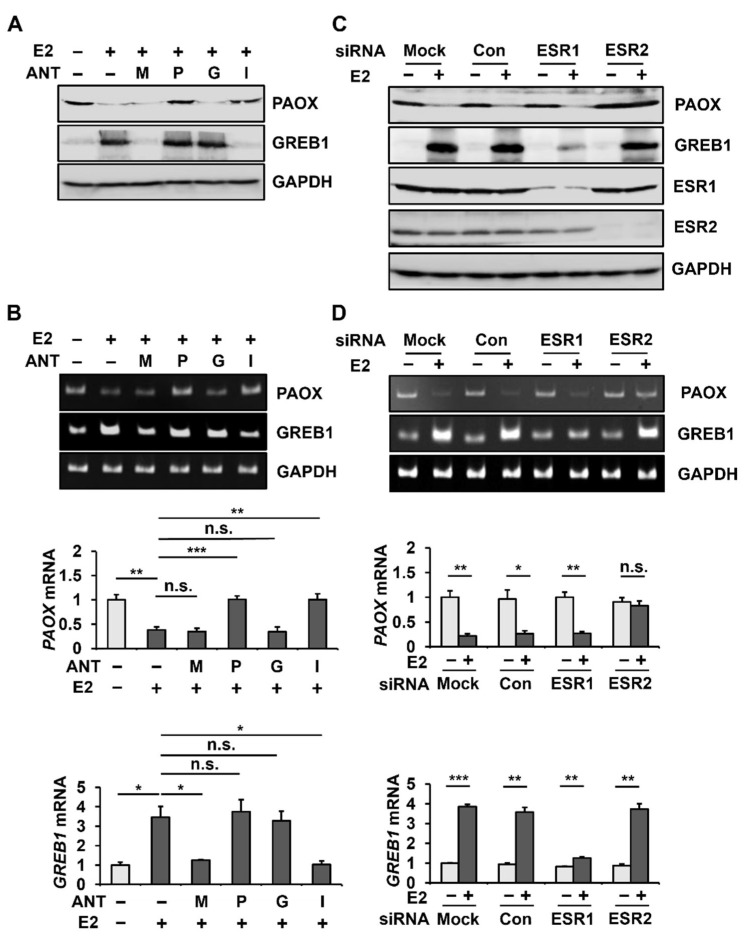
E2-dependent PAOX downregulation is mediated by ESR2. MCF-7 cells were incubated in 10% CS-FBS in PRF-DMEM in the presence of DMSO (-; 100 µM), ESR1 antagonist MPP (M; 100 µM), ESR2 antagonist PHTPP (P; 100 µM), G protein-coupled ER antagonist G-15 (G; 100 µM), or non-specific ESR antagonist ICI 182,780 (I; 100 µM) for 5 h. MCF-7 cells were then incubated in 10% charcoal-stripped FBS in PRF-DMEM, with or without E2. Protein levels of PAOX, GREB1, and GAPDH were evaluated using Western blotting (**A**). The mRNA levels of *PAOX*, *GREB1*, and *GAPDH* were evaluated by conventional (top) and quantitative (bottom) PCR (**B**). MCF-7 cells were transfected with siRNA for Con (scrambled), *ESR1*, or *ESR2* knockdown for 5 h. Protein levels of PAOX, ESR1, ESR2, GREB1, and GAPDH were evaluated using Western blotting (**C**). The mRNA expression levels of *PAOX*, *GREB1*, and *GAPDH* were evaluated by conventional (top) and quantitative (bottom) PCR (**D**). Data are shown as the mean ± S.D. (*n* = 3), normalized to *GAPDH* expression. * *p* < 0.05; ** *p* < 0.01; and *** *p* < 0.001 versus control.

To understand the estrogen receptor signaling pathway involved in E2-mediated PAOX downregulation, we analyzed E2-mediated PAOX expression in MCF-7 cells in the presence of MPP (antagonist of ESR1 (also known as ERα)), PHTPP (antagonist of ESR2 (also known as ERβ)), G-15 (antagonist of G Protein-Coupled Estrogen Receptor 1 (also known as GPER1)), and ICI 182,780 (non-specific ESR antagonist). E2-mediated upregulation of GREB1, which is known to involve ESR1 [31], was blocked by MPP and ICI 182,780. However, the E2-dependent downregulation of PAOX was hampered by PHTPP and ICI 182,780 but was not affected by MPP and G-15 (Figure 3A). E2-dependent decrease in *PAOX* mRNA levels was also blocked by PHTPP and ICI 182,780 but not MPP and G-15 (Figure 3B). These results suggest that E2 reduces PAOX expression through ESR2 in MCF-7 cells. 

To clearly define the estrogen receptor involved in the E2-dependent downregulation of PAOX expression, we analyzed E2-mediated PAOX expression in MCF-7 cells with *ESR1* or *ESR2* knockdown using siRNA. The transfection of *ESR1* or *ESR2* siRNA into MCF-7 cells reduced the protein expression of ESR1 and ESR2, respectively (Figure 3C). *ESR2* knockdown alleviated the decrease in PAOX induced by E2, while mock transfection or transfection with scrambled siRNA (control) or *ESR1* siRNA did not alter the E2-mediated decrease in PAOX (Figure 3C). As previously reported [32], *ESR1* knockdown severely blocked the increase in GREB1 by E2. In addition, it was also confirmed that E2-mediated *PAOX* repression and *GREB1* induction were inhibited by *ESR2* knockdown and *ESR1* knockdown at the mRNA level, respectively (Figure 3D).

### 2.3. E2 Reduces the Activity of the PAOX Promoter in an ESR2-Dependent Manner

**Figure 4:**
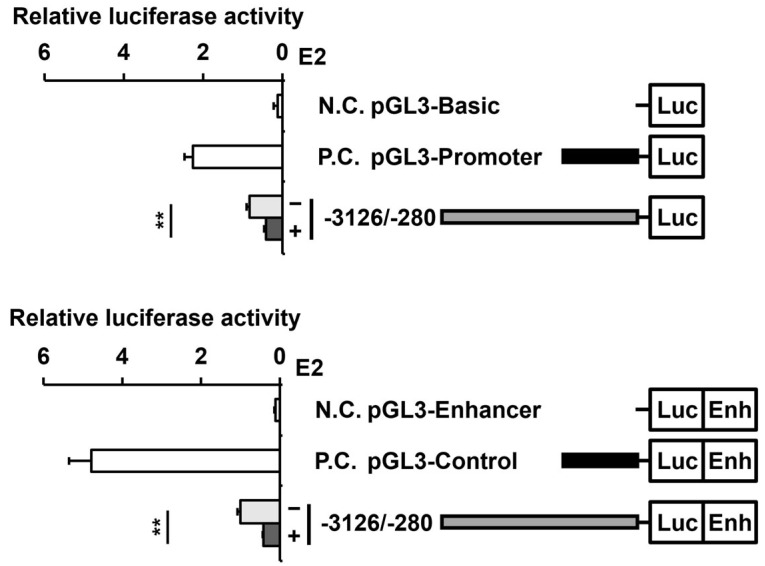
Activities of the luciferase reporters driven by the 5′-flanking regions of the *PAOX* gene are repressed by E2. MCF-7 cells were co-transfected with pRL-TK and promoterless pGL3-Basic (negative control), pGL3-Promoter containing SV40 promoter (positive control), or pGL3-*PAOX* promoter (−3126/−280) containing the 5′-flanking sequence from −3126 to −280 (−1 from upstream of ATG) or with promoterless pGL3-Enhancer (negative control), pGL3-Control containing SV40 promoter and enhancer (positive control), or pGL3-Enhancer-*PAOX* promoter (−3126/−280). MCF-7 cells transfected with the pGL3-Basic-*PAOX* promoter (−3126/−280) or pGL3-Enhancer-*PAOX* promoter (−3126/−280) were treated with or without E2 for 24 h. Cell lysates were subjected to luciferase assays. Luminescence was measured at 560 nm for firefly luciferase and 480 nm for *Renilla* luciferase. Data are shown as the mean ± S.D. (*n* = 3), normalized to *Renilla* luciferase activity. ** *p* < 0.01 versus the *PAOX* promoter activity in the absence of E2.

To understand whether the decrease in *PAOX* mRNA and protein levels by E2 is due to the decrease in *PAOX* transcriptional activity, we established constructs in which firefly luciferase cDNA was fused to the *PAOX* promoter (nucleotide positions −3126 to −280 from the start codon), and changes in *PAOX* promoter activity by E2 treatment in MCF-7 cells were measured by luciferase activity. Luciferase activity is driven by the *PAOX* promoter without an enhancer (pGL3-Basic-*PAOX* promoter (−3126/−280)) and was reduced to 49.9 ± 5.7% by treatment with E2 (Figure 4). Luciferase activity driven by the *PAOX* promoter in the presence of the SV40 enhancer (pGL3-Enhancer-*PAOX* promoter (−3126/−280)) was also reduced to 41.3 ± 1.6% by E2, a slightly larger difference in comparison to the absence of the SV40 enhancer. Therefore, it was confirmed that *PAOX* promoter activity is suppressed by E2. For subsequent analysis, we used a *PAOX*-promoter-driven luciferase construct containing the SV40 enhancer, where the effect of E2 can be better observed. 

We then analyzed whether the reduction in *PAOX* promoter activity by E2 was ESR2-dependent by treatment with an ESR antagonist and siRNA in a *PAOX* promoter (−3126/−280)-driven reporter construct. As expected, the reduction in *PAOX* promoter activity by E2 was restored by PHTPP and ICI 182,780 but was not significantly altered by MPP and G-15 (Supplementary Figure S1A). In addition, transfection with *ESR2* siRNA reversed the decrease in PAOX promoter activity by E2, but transfection with *ESR1* siRNA did not (Supplementary Figure S1B).

### 2.4. Two AP-1 Sites within the PAOX Promoter Are Involved in the E2-Mediated PAOX Repression

**Figure 5:**
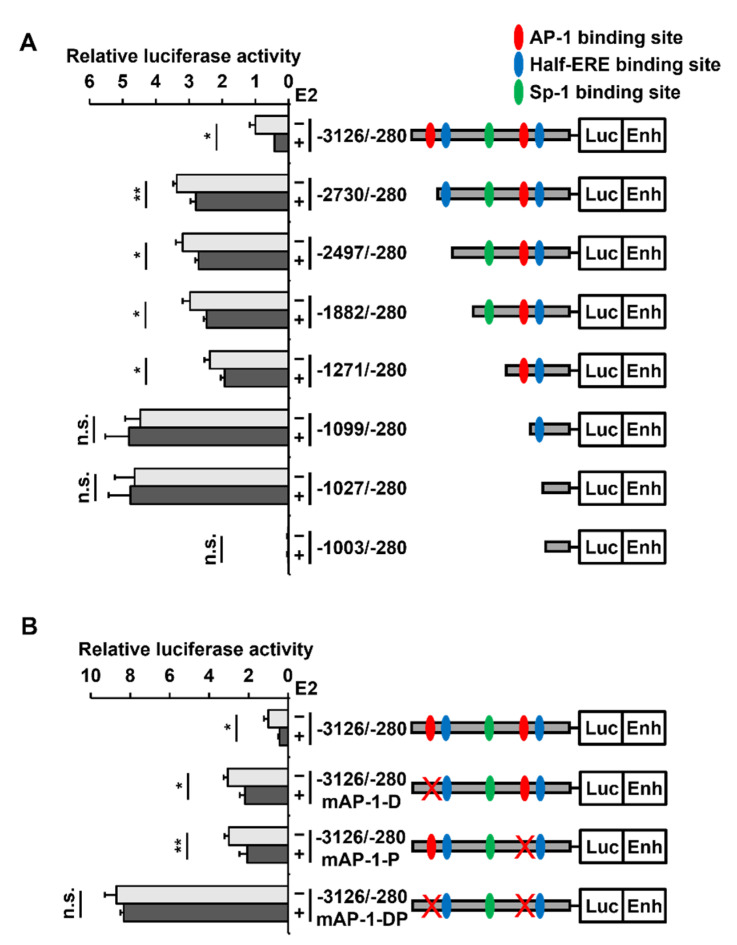
Repression of the *PAOX* promoter activity by E2 depends on two AP-1 binding sites of the *PAOX* promoter. MCF-7 cells were co-transfected with pRL-TK and pGL3-Enhancer-*PAOX* promoter (-3126/-280), or its derivatives, including serial 5′ deletion of the *PAOX* promoter; (−2730/−280), (−2730/−280), (−2497/−280), (−1882/−280), (−1271/−280), (−1099/−280), (−1027/−280), and (−1003/−280) (**A**) or harboring mutations in the AP-1 binding sites of the *PAOX* promoter; mAP-1-D with a mutation in the distal AP-1 site; mAP-1-P with a mutation in the proximal AP-1 site; and mAP-1-DP with mutations in both distal and proximal AP-1 sites (**B**). Luciferase assays were performed as previously described. Data are shown as the mean ± S.D. (*n* = 3), normalized to *Renilla* luciferase activity. * *p* < 0.05 and ** *p* < 0.01 versus the promoter activity in the absence of E2.

Using a transcription factor binding site search program (Jaspar database), two AP-1 at −2747/−2741 (TGATTCA) and −1158/−1152 (GGAGTCA), two half-ERE at −2516/−2511 (AGGTCA) and −1059/−1054 (AGGTCA), and one SP-1 at −1325/−1317 (TCGGGTGGGT) sites were identified in the *PAOX* promoter (−3126/−280) (Figure 5A). Luciferase analysis of pGL3-Enhancer–*PAOX* promoter (−3126/−280) derivatives with serial 5’-truncation of the *PAOX* promoter revealed that when the −3126/−2730 and −1271/−1099 regions including each of the two AP-1 binding sites in the *PAOX* promoter were deleted, promoter activity was increased gradually. In addition, these deletions gradually reduced the extent of the E2-dependent decrease in *PAOX* promoter (−3126/−280) activity. The −1099/−280 and −1027/−280 fragments of the *PAOX* promoter lacking two AP-1 sites no longer showed the E2-dependent decrease in promoter activity. The −1003/−280 fragment of the *PAOX* promoter did not show any promoter activity. These findings suggested that the two putative half-ERE sites and a putative SP-1 binding site in the *PAOX* promoter were not involved in the E2-dependent repression of the *PAOX* promoter. 

To confirm the importance of AP-1 sites in E2-induced PAOX repression, site-directed mutagenesis at each of the two AP-1 binding sites, and subsequently at both the sites in the *PAOX* promoter, was performed, and the promoter activity of the reporter constructs was then measured. Luciferase analysis of the constructs showed that the repression of the *PAOX* promoter by E2 was reduced when the distal or proximal AP-1 binding site was mutated. In addition, when both AP-1 binding sites were mutated, the *PAOX* promoter activity was no longer altered by E2 (Figure 5B).

### 2.5. ESR2 Binds to the AP-1 Binding Sites of the PAOX Promoter

**Figure 6:**
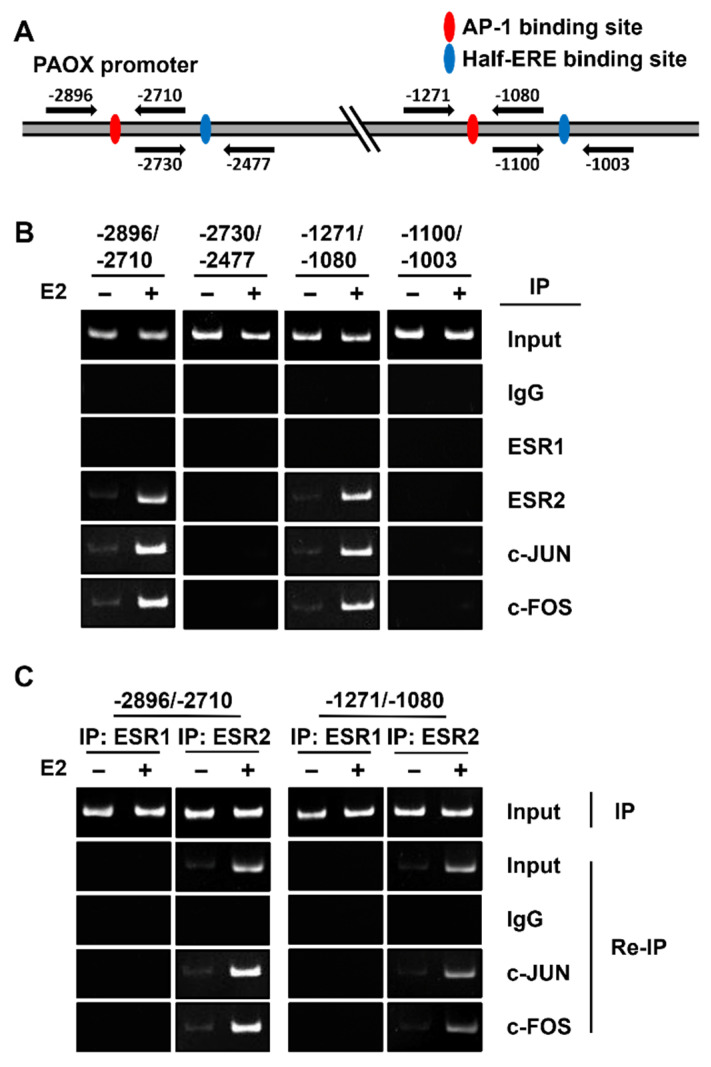
ESR2 interacts with AP-1, which binds to the AP-1 binding sites in the *PAOX* promoter in an E2-dependent manner. Primer pairs for PCR amplification of the AP-1 binding site and the half-ERE binding site in the *PAOX* promoter are indicated by arrows (**A**). The regions for PCR were −2896 to −2710 for the distal AP-1 site, −1271 to −1080 for the proximal AP-1 site, −2730 to −2477 for the distal putative half-ERE site, and −1100 to −1003 for the proximal putative half-ERE sites. Chromatin extracts were prepared from MCF-7 cells treated with or without E2, and immunoprecipitation of the extracts was performed using the indicated antibodies for ChIP assays (**B**). Chromatin extracts were immunoprecipitated using an anti-ESR1 antibody or anti-ESR2 antibody and then with control anti-IgG, anti-c-JUN, or anti-c-FOS antibody for Re-IP assays (**C**). PCR was performed with the above primer pairs, using DNA extracted from the immunoprecipitates as templates. PCR products were analyzed using 5% PAGE gels.

We showed that both ESR2 and AP-1 binding sites of the *PAOX* promoter are important for the E2-dependent repression of *PAOX* promoter activity. Therefore, the binding of c-JUN and c-FOS, constituting AP-1, and of ESR2 to the two AP-1 binding sites of the *PAOX* promoter was examined using chromatin immunoprecipitation (ChIP) analysis. ChIP reactions involving ESR1 and two putative half-ERE binding sites were used as negative controls. PCR products encompassing each of the two AP-1 binding sites were observed in all samples immunoprecipitated with the ESR2, c-JUN, or c-FOS antibodies in an E2-dependent manner but not in the sample immunoprecipitated with the ESR1 antibody (Figure 6A). In addition, no PCR products for the two half-ERE binding sites were observed in samples precipitated with ESR1, ESR2, c-JUN, or c-FOS antibodies. Therefore, c-JUN, c-FOS, and ESR2 were found to specifically bind to each of the two AP-1 binding sites in the *PAOX* promoter.

To analyze whether ESR2 binds to the AP-1 (c-JUN and c-FOS) bound to each of the two AP-1 binding sites of the *PAOX* promoter in an E2-dependent manner, Re-Immunoprecipitation (Re-IP) analysis was performed by immunoprecipitation of chromatin extracts first with anti-ESR1 or anti-ESR2 antibodies and secondarily with c-JUN or c-FOS antibodies. PCR products for each of the two AP-1 binding sites were observed in immunoprecipitates with the ESR2 antibody and then the c-FOS or c-JUN antibody in an E2-dependent manner (Figure 6B). PCR products for AP-1 binding sites were not detected in any immunoprecipitates with the ESR1 antibody. Therefore, we concluded that ESR2 binds to AP-1 (c-JUN and c-FOS) and is bound to each of the two AP-1 binding sites of the *PAOX* promoter in an E2-dependent manner.

## 3. Discussion

Herein, we examined changes in the expression of polyamine metabolic enzymes at the RNA level in ER-positive MCF-7 breast cancer cells, with and without the influence of E2. We found that E2 upregulates the mRNA levels of anabolic enzymes of polyamine metabolism, including *AMD1*, *ODC1*, and *SRM* but not *SMS,* which catalyzes the conversion of spermidine to spermine, and downregulates the mRNA levels of catabolic enzymes such as *SAT1*, and *PAOX* but not *SMOX*, which catalyzes the direct conversion of spermine to spermidine. We also found that, among polyamine metabolic enzymes, *PAOX* was most significantly altered by E2 in MCF-7 cells at the mRNA level. Furthermore, *PAOX* was reduced by E2 at both the protein and mRNA levels. As *PAOX* converts N1-acetylspermine and N1-acetylspermidine produced by SAT1 to spermidine or putrescine by oxidation, decreased *PAOX* expression results in increased polyamine and decreased H_2_O_2_ levels [33,34].

Estrogen transmits signals through three ESRs, namely, ESR1, ESR2, and the G-protein-coupled estrogen receptor 1 (GPER1) [35,36,37]. Once E2 binds to ESR1 or ESR2 in the cytoplasm, the ESRs undergo a structural change that induces receptor dimerization [38]. These E2–ESR complexes translocate to the nucleus and play a role in gene regulation by binding to the estrogen response element (ERE)-binding sites or transcription factors such as AP-1 and SP-1 [39,40,41,42]. In contrast to the ESR1 and ESR2, the GPER1 shows characteristics of G-protein-coupled receptors that act via the activation of the heterotrimeric G protein [43]. The GPER1 is anchored in the plasma membrane, and unlike nuclear estrogen receptors, signaling pathways involving GPER1 activation occur through a variety of secondary messengers via G-protein activation [44].

ESRs can regulate the expression of target genes by directly binding to EREs identified in estrogen-responsive promoters [32]. In addition, ESRs can regulate gene expression through protein–protein interactions with other transcription factors, such as the SP-1 and AP-1 [39,40,41], and co-regulators that have chromatin remodeling functions [45,46]. Approximately one-third of estrogen target genes in humans do not have an ERE or an ERE-like region [39]. 

ESRs can activate or repress the transcription of target genes. Molecular mechanisms that increase target gene expression through E2-ESR have been studied more than those that repress expression [47]. However, because nearly 70% of E2-regulated genes are repressed in MCF-7 cells [48], estrogen-mediated repression of target genes appears to be more common than activation. In estrogen-stimulated MCF-7 cells, the 6024 ESR1 and 9702 ESR2 binding sites, where the ESR1, ESR2, or both ER subtypes can bind, were identified using ChIP-Seq analysis [49]. Based on these results, it is expected that ESR2 is involved in the regulation of more genes than ESR1, at least in MCF-7 cells.

We established that ESR2 is responsible for E2-dependent *PAOX* expression repression and reporter assays using antagonists and siRNAs; however, the mechanism by which the E2–ESR2 complex downregulates PAOX expression was unclear. When we deleted the two AP-1 binding sites of the *PAOX* promoter, *PAOX* promoter activity in the absence of E2 gradually increased, consistent with a previous report [50]. More importantly, the deletion or mutation of the two AP-1 binding sites of the *PAOX* promoter gradually leads to the loss of E2-dependent promoter activity repression, while the deletion of two putative half-ERE and SP-1 binding sites did not affect promoter activity. ChIP and Re-IP assays revealed that the ESR2 as well as the c-FOS, and the c-JUN bound to each of the two AP-1 binding sites in the *PAOX* promoter, but the ESR2 did not bind to either of the two putative half-ERE sites. We confirmed that the E2–ESR2 complex binds to AP-1 bound to the two AP-1 binding sites of the *PAOX* promoter, thereby repressing *PAOX* transcription in MCF-7 cells.

The molecular mechanism of target gene downregulation by E2–ESR2 has been reported previously. For example, *TNFAIP1* is downregulated by the binding of the E2–ESR2 directly to the ERE site on its promoter in primary hippocampal cells [51]. In addition, ESR1 expression is repressed by the indirect binding of E2–ESR2 to SP-1 in MCF-7 cells [52]. As a novel finding, we showed that the E2–ESR2 complex downregulates the expression of PAOX by binding to the AP-1 bound to AP-1 binding sites. Therefore, to the best of our knowledge, our finding is the first example of E2 repressing a target gene through ESR2–AP-1 interaction, independent of ERE.

In this study, we found that E2 upregulated the expression of most anabolic enzymes and downregulated the expression of most catabolic enzymes involved in polyamine metabolism. In addition, we demonstrated that PAOX expression, which was most significantly altered by E2 in MCF-7 cells, was repressed by the binding of the E2–ESR2 complex to AP-1 bound to the two AP-1 binding sites of the *PAOX* promoter. 

Our data suggest that an increase in estrogen level would lead to an increase in the expression of polyamine anabolic enzymes, including the *AMD1*, *ODC1*, and *SRM*, and a decrease in the expression of polyamine catabolic enzymes, including *SAT1* and *PAOX*, which leads to an increase in polyamine levels. This finding is consistent with a previous report that polyamine levels were increased upon E2 treatment in MCF-7 cells [53]. This estrogen-dependent change in polyamine levels may help to understand the hormone therapy of breast cancer and side effects of menopause, from the perspective of polyamine. A selective estrogen receptor modulator (SERM), such as tamoxifen, can reduce the incidence of hormone-receptor-positive breast cancer and the likelihood of cancer recurrence [54]. SERM treatment reduces polyamine levels in MCF-7 cells [55]. It is well documented that polyamines play a role in tumorigenesis, at least by increasing cell proliferation and suppressing apoptosis [4]. For example, the ODC1 inhibitor α-difluoromethylornithine is known to prevent the growth-promoting effect of E2 [56,57]. Moreover, sensitivity to the growth-promoting effects of E2 was reduced by about a third in MCF-7 cells overexpressing ODC1 compared with vector-transfected cells [58]. These results suggest that elevated polyamine levels contribute to estrogen-induced oncogenic phenotypes in breast cancer cells. In addition, a decrease in estrogen during menopause leads to a decrease in polyamine levels. Polyamine reduction is a common occurrence in postmenopausal women and has many clinical implications [59,60,61,62]. For example, polyamines effectively prevent bone loss in ovariectomized mice [20]. In addition, polyamine reduction leads to increased H_2_O_2_ and ROS levels, which, as previously mentioned, contribute to inflammatory diseases [63]. Therefore, we propose that preventing polyamine decline, for example by repressing or inhibiting PAOX, could be a potential treatment for alleviating postmenopausal symptoms.

## 4. Methods and Materials

### 4.1. Reagents and Antibodies

The ESR1 antagonist 1,3-*Bis*(4-hydroxyphenyl)-4-methyl-5-[4-(2-piperidinylethoxy)phenol]-1*H*-pyrazole dihydrochloride (MPP), ESR2 antagonist 4-[2-Phenyl-5,7-*bis*(trifluoromethyl)pyrazolo [1,5-*a*]pyrimidin-3-yl]phenol (PHTPP), the GPER1 antagonist (3aS*,4R*,9bR*)-4-(6-Bromo-1,3-benzodioxol-5-yl)-3a,4,5,9b-3*H*-cyclopenta[*c*]quinoline (G-15), and the non-specific ESR antagonist 7α,17β-[9-[(4,4,5,5,5-Pentafluoropentyl)sulfinyl]nonyl]estra-1,3,5(10)-triene-3,17-diol (ICI 182,780) were purchased from Tocris (Bristol, UK). The water-soluble β-estradiol (E2) and 2-hydroxypropyl-β-cyclodextrin (carrier for β-estradiol) were purchased from Sigma-Aldrich (St. Louis, MO, USA). The following antibodies were used: anti-PAOX (MBS3211539), obtained from MyBioSource (San Diego, CA, USA); anti-ESR1 (sc-8002) and anti-ESR2 (sc-373885), obtained from Santa Cruz Biotechnology (Cambridge, UK); anti-GREB1 (ab-72997), obtained from Cell Signaling (Danvers, MA, USA); anti-c-JUN (2250S) and anti-c-FOS (9165S), obtained from Abcam (Cambridge, UK); anti-GAPDH (AbC-2003), obtained from AbClone (Seoul, Korea); and the horseradish peroxidase-conjugated goat anti-mouse IgG and goat anti-rabbit IgG described previously [64].

### 4.2. Cell Culture

The MCF-7 cells were grown in DMEM (Hyclone, South Logan, UT, USA) supplemented with 10% fetal bovine serum (FBS; Gibco of Thermo Fisher Scientific, Waltham, MA, USA), 100 U/mL of penicillin, and 100 μg/mL of streptomycin at 37 °C under 5% CO_2_ and 95% air.

### 4.3. RNA Isolation and PCR

The total RNA was isolated from MCF-7 cells using TRIZOL (Invitrogen, Thermo Fisher Scientific), as described previously [65]. The cDNA mixtures were synthesized from the total RNA by reverse transcription-PCR using oligo dT_15_ primers and the AMV RT system (Promega, Madison, WI, USA) according to the manufacturer’s instructions. Conventional PCR was performed in a final volume of 10 μL containing 1 pM each of the 5′ primer and 3′ primer, 0.2 mM dNTPs, 1× *Taq* PCR buffer, 50 U/mL *Taq* polymerase, and cDNAs synthesized from 0.1 μg total RNA. PCR was performed with 25–30 cycles; the cycling conditions were as follows: denaturation at 94 °C for 30 s, annealing at the appropriate annealing temperature (Supplementary Table S1) for 30 s, and extension at 72 °C for 30 s. The PCR products were detected by a 5% PAGE analysis and visualized by ethidium bromide staining. Real-time PCR was conducted using a QuantiTect SYBR Green PCR kit (Qiagen, Hilden, Germany) and QuantStudio 3 Real-Time PCR system (Applied Biosystems, Foster City, CA, USA). The expression of the target genes was normalized to that of *GAPDH* expression.

### 4.4. Western Blotting

The cells were harvested using an SDS sample buffer (50 mM Tris-HCl, pH 6.8, 2% SDS, 0.1% bromophenol blue, and 10% glycerol) on ice for 15 min and centrifuged at 18,000 × *g* for 15 min after brief sonication. The cell lysate samples were boiled after the addition of 100 mM β-mercaptoethanol for 4 min and resolved by SDS-PAGE. Proteins in the gel were blotted onto polyvinylidene fluoride (PVDF) membranes (Millipore, Billerica, MA, USA). The blots were incubated with the indicated primary and secondary antibodies. Immunoreactive signals were detected using the Immobilon Western HRP substrate (Millipore, Billerica, MA, USA) and an LAS-3000 detector (Fujifilm, Tokyo, Japan) as previously described [66].

### 4.5. Treatment of E2 to MCF-7 Cells

The subconfluent MCF-7 cells were incubated in phenol-red free DMEM (PRF-DMEM; HyClone) containing 10% charcoal-stripped FBS (Scipak Lifesciences, Arlington, VA, USA) for 24 h. The medium was replaced with a fresh medium containing water-soluble β-estradiol (10 nM E2 in the presence of 35.1 nM 2-hydroxypropyl-β-cyclodextrin) or 2-hydroxypropyl-β-cyclodextrin (35.1 nM).

### 4.6. Transfection of siRNAs

The ON-TARGETplus Human *ESR1* and *ESR2* siRNAs (600 pM siRNA/100 mm plate; Dharmacon, Lafayette, CO, USA) (600 pM siRNA/100 mm plate) were transfected into MCF-7 cells using Lipofectamine 2000 (Thermo Fisher Scientific).

### 4.7. Prediction of Transcription Factor-Binding Site

The transcription factor-binding sites on the *PAOX* promoter were searched using CiiiDER, an integrated computational toolkit [67], and the transcription factor-binding site database from JASPAR (http://jaspar.cgb.ki.se/, accessed on 23 September 2021).

### 4.8. Cloning of the Human PAOX Promoter in Luciferase Reporter Vectors

To generate a luciferase reporter construct for the human *PAOX* promoter (GenBank accession no. NM_152911.4), a fragment of the *PAOX* promoter −3126/−280 (the first nucleotide upstream of the start codon is referred to as −1) was PCR-amplified using genomic DNA from human foreskin fibroblasts (as previously described [68]) as a template with PrimeSTAR^®^ GXL DNA polymerase (TaKaRa, Shiga-ken, Japan). The deletion products were PCR-amplified using the *PAOX* promoter (−3126/−280) as a template, using the indicated primer pairs (Supplementary Table S2). The PCR products were cloned into pGL3-Basic or pGL3-Enhancer luciferase reporter vectors digested with Acc65I and HindIII (Promega, Madison, WI, USA) using the In-Fusion Cloning system (Takara). Luciferase reporter constructs harboring the human *PAOX* promoter were confirmed to be error-free by DNA sequencing.

### 4.9. Dual-Luciferase Reporter Assay

The transfection of the luciferase constructs into MCF-7 cells was performed using Lipofectamine 2000. The MCF-7 cells were plated in 96-well plates at a density of 5 × 10^4^ cells/well and incubated in 100 μL of DMEM supplemented with 10% FBS for 12 h. Promoterless pGL3-Basic (0.11 μg) or pGL3-Basic-*PAOX* promoter constructs (0.18 μg) encoding firefly luciferase driven by *PAOX* promoter, or promoterless pGL3-Enhancer (0.12 μg) or pGL3-Enhancer-*PAOX* promoter constructs (0.19 μg of pGL3-Enhancer-*PAOX* promoter(-3126/-280) or the amount corresponding to the size of each construct) and pRL-TK encoding *Renilla* luciferase driven by herpes simplex virus thymidine kinase promoter (0.02 μg; Promega, Madison, WI, USA) in 25 μL Opti-MEM (Gibco/Thermo Fisher Scientific) were incubated with Lipofectamine 2000 (0.75 μL) and diluted in 25 μL Opti-MEM for 30 min at room temperature. Cells in Opti-MEM were treated with this mixture for 5 h and then replaced with DMEM supplemented with 10% FBS for 12 h. The cells were then incubated with PRF-DMEM supplemented with 10% charcoal-stripped FBS for 24 h. Next, the cells were treated with or without E2 in PRF-DMEM supplemented with 10% charcoal-stripped FBS for 24 h. Luciferase activity was measured using the dual-luciferase reporter assay system (Promega), and the firefly luciferase activity in transfected cells was normalized to that of the *Renilla* luciferase.

### 4.10. Site-Directed Mutagenesis (SDM)

Mutations in the distal and proximal AP-1 sites in the *PAOX* promoter were introduced by overlap extension PCR mutagenesis using the PrimeSTAR^®^ GXL DNA polymerase. The sequences of the primer pairs used are listed in Supplementary Table S3. To generate a mutation in the distal AP-1 site, two primary PCR products with Primer −3126-F and mAP-1-D-R and with Primer mAP-1-D-F and −280-R using the pGL3-Enhencer-*PAOX* promoter (−3126/−280) as a template were obtained. Similarly, to generate a mutation in the proximal AP-1 site, two primary PCR products with Primer −3126-F and AP-1-P-R and with Primer mAP-1-P-F and −280-R using the pGL3-Enhencer-*PAOX* promoter (−3126/−280) as a template were obtained. In addition, to generate double mutations in the proximal and distal AP-1 sites, two primary PCR products with Primer −3126-F and AP-1-P-R and Primer mAP-1-P-F and −280-R using the pGL3-Enhencer-*PAOX* promoter (−3126/−280)-mAP-1-D as a template were obtained. Then, a second PCR was performed to create an overlap-extension PCR product using a mixture of PCR products for distal AP-1 site mutation or proximal AP-1 site mutation as a template, as well as Primer −3126 and −280. Overlap-extended PCR products harboring mutations in the distal or proximal AP-1 site or double mutations in the distal and proximal AP-1 sites were cloned into the pGL3-Enhancer vector cleaved with Acc65I and HindIII to generate the pGL3-Enhancer-*PAOX* promoter (−3126/−280)-mAP-1-D, -mAP-1-P, and -mAP-1-DP, respectively. Luciferase reporter constructs with mutations in AP-1 sites of the *PAOX* promoter were confirmed to be error-free by DNA sequencing.

### 4.11. ChIP and Re-IP

ChIP was performed as previously described [69], with minor modifications. The MCF-7 cells were treated with E2 or vehicle for 24 h and fixed with 1% formaldehyde at room temperature for 10 min. The reaction was stopped by adding 0.125 M glycine and washing twice after 5 min with ice-cold PBS. The cells were lysed for 10 min by adding a cell lysis/wash buffer (150 mM NaCl, 50 mM Tris-HCl, pH 7.5, 5 mM EDTA, 0.5% NP-40, 1.0% Triton X-100) at 4 °C and a protease-inhibitor cocktail (Sigma-Aldrich, MO, USA). Then, cells were centrifuged at 1500 rpm at 4 °C for 5 min, and the pellet containing the chromatin fraction was lysed in a shearing buffer (1% SDS, 10 mM EDTA, 50 mM Tris-HCl pH 8.0) and protease inhibitor cocktail. The chromatin extracts containing genomic DNA were sonicated on ice to obtain an average fragment size of 500 bp. The supernatant was diluted 9 times with a diluted buffer (0.01% SDS, 1.1% Triton X-100, 1.2 mM EDTA, 16.7 mM Tris-HCl, pH 8.1, 167 mM NaCl) and subsequently immunoprecipitated using antibodies and Protein G-agarose (Invitrogen) at 4 °C. The immunoprecipitates were washed once with cold cell lysis/wash buffer and three times with cold ethanol. DNA purification followed by protein degradation was performed using Chelex 100 (Bio-Rad, Hercules, CA, USA), following the manufacturer’s instructions. The PCR was performed in 30 cycles under the following conditions: denaturation at 94 °C for 30 s, annealing at an indicated annealing temperature (Supplementary Table S4) for 30 s, and extension at 72 °C for 30 s. The Re-IP assay was performed as previously described [70] with minor modifications. The immunoprecipitated antibody–protein–DNA complexes were washed twice with the cell lysis/wash buffer and eluted by incubating for 30 min at 37 °C in a Re-IP elution buffer (TE buffer (10 mM Tris-HCl, pH 8.0, 1 mM EDTA), 2% SDS, 15 mM DTT). After centrifugation, the supernatant was diluted 20 times using a dilution buffer and individually re-immunoprecipitated with each secondary antibody at 4 °C. The immune complexes were incubated with protein G-agarose beads for 2 h at 4 °C. The DNA was purified from the secondary immune complex using Chelex 100. The PCR was performed using the extracted DNA as described above.

### 4.12. Statistical Analyses

All data are presented as a mean ± standard deviation of at least three independent experiments. Statistical significance was analyzed using the unpaired two-tailed Student’s t-test. A *p*-value < 0.05 was considered statistically significant.

## 5. Conclusions

In this study, we showed that *PAOX* was significantly altered among polyamine metabolism-related enzymes upon E2 treatment in MCF-7 cells. We demonstrated that PAOX expression was downregulated by E2 via ESR2. The ESR2 binds to the AP-1 complex bound to two AP-1 binding sites in the *PAOX* promoter. The E2–ESR2–AP-1 complex binds to the *PAOX* promoter and downregulates PAOX expression. Based on our findings, we suggest that E2-dependent inhibition of PAOX is at least one cause of polyamine decline during menopause

## Data Availability

Authors can confirm that all relevant data are included in the article.

## Supplementary Materials

The Supplementary Materials are available online at https://www.mdpi.com/article/10.3390/ijms23147521/s1.
