# Supplementary material for: Polyamine Oxidase Expression Is Downregulated by 17β-Estradiol via Estrogen Receptor 2 in Human MCF-7 Breast Cancer Cells
Source: Int J Mol Sci. 2022 Jul 7;23(14):7521. doi: 10.3390/ijms23147521 (PMC9317983; doi:10.3390/ijms23147521)
Supplement: Supplementary file 1 [file ijms-23-07521-s001.zip › ijms-1774133-supplementary.pdf]

Supplementary Materials

Polyamine oxidase expression is downregulated by 17β-estradiol via estrogen receptor 2 in human MCF-7 breast cancer cells

Jin Hyung Kim[1] and Seung-Taek Lee[1,*]

[1] Department of Biochemistry, College of Life Science and Biotechnology, Yonsei University, Seoul 03722, Korea; qnt1313@yonsei.ac.kr (J.H.K.)

* Correspondence: stlee@yonsei.ac.kr; Tel.: +82-2-2123-2703

Supplementary Table S1. Primer sequences used for reverse transcription-polymerase chain reaction of *AMD1*, *ODC1*, *SAT1*, *SMOX*, *SMS*, *SRM*, *PAO*X, *GREB1,* and *GAPDH* mRNAs.

**Primer Name**[1]	**Nucleotide Sequence**	**Nucleotide Position**	**Annealing Temp. (℃)**	**GenBank Number**
AMD1-F	5′-GTGGTTGGAACACTGTTTGACT-3′	2945–2966	55	NM_001634.6
AMD1-R	5′-AGGTGCTGTGCTCATTACAGA-3′	3056–3036		
OCD1-F	5′-CGGATTGTTGAGCGCTGTGACC-3′	1427–1448	60	NM_002539.3
OCD1-R	5′-GGCAGCAGCAACAGTGTAAGCG-3′	1516–1495		
SAT1-F	5′-GCAGCAGCATGCACTTCTTGGTA-3′	544–566	60	NM_002970.4
SAT1-R	5′-AGTCTCCAACCCTCTTCACTGGA-3′	646–624		
SMOX-F	5′-ATGCAGGTGCTGTTTTCCGGTGA-3′	1710–1732	65	NM_175839.3
SMOX-R	5′-GGTACATCTCAATGAGGCGGGC-3′	1818–1797		
SMS-F	5′-ATCTGACAGAAGCACTGTCGCTC-3′	1075–1097	55	NM_004595.5
SMS-R	5′-TATGAAGGGACACAGACGATCTCC-3′	1168–1145		
SRM-F	5′-CAGCAAGAACCCGAGCACGAACT-3′	828–850	60	NM_003132.3
SRM-R	5′-GCAAACTCGGGCAGCACAAAGG -3′	956–935		
PAOX-F	5′-AAGAGCGTCCTGCGGTCTCG-3′	1313–1332	60	NM_152911.4
PAOX-R	5′-CGTCCGTCGTGGAGTAAAACGT-3′	1501–1484		
GREB1-F	5′-GCTGGAAAGAGCTAGAAGCACAGTTC-3′	7722–7747	65	NM_014668.4
GREB1-R	5′-TGGCATTGAGGGTAGGCAAG -3′	7813–7794		
GAPDH-F	5′-ACTGCTTAGCACCCCTGGCCA-3′	540–560	57	NM_002046.7
GAPDH-R	5′-TTGGCAGTGGGGACACGGAAG-3′	792–772		

[1] F: forward primer and R: reverse primer

Supplementary Table S2. Primer sequences used for the cloning of PAOX promoter-reporter constructs.

**Primer Name**[1]	**Nucleotide Sequence**[2]	**Annealing Temp. (℃)**
-3126-F	5′-**TCTATCGATA***GGTACC*TGAGGTCAGGTGTTCGAGACC-3′	65
-2730-F	5′-**TCTATCGATA***GGTACC*ATGGGTAGTTGCCACCTTG-3′	65
-2497-F	5′-**TCTATCGATA***GGTACC*GTGCTATTGGATTCAGGC-3′	65
-1882-F	5′-**TCTATCGATA***GGTACC*TGAAAACAGGGCAGCAGTC-3′	65
-1271-F	5′-**TCTATCGATA***GGTACC*GTTCCCCATGGCCTGGAG-3′	65
-1099-F	5´-**TCTATCGATA***GGTACC*GTTGGCTAGGGAGTGATGG-3´	65
-1027-F	5′-**TCTATCGATA***GGTACC*GGGACGAGAGGGAATCAAAGG-3′	65
-1003-F	5´-**TCTATCGATA***GGTACC*GTAAGACACGGCTCAGGAG-3´	65
-280-R	5′-**CCGGAATGCC***AAGCTT*GGGGCCGGGCCGAGCCCCAC-3′	65

[1] Each primer was named based on the 5′-end nucleotide position of the PAOX promoter that annealed to each primer. The 1st nucleotide upstream of the start codon was referred to as -1. F: forward primer and R: reverse primer.

[2] Sequences annealed to the pGL3 vector and corresponding to the restriction site (GGTACC or AAGCTT) are shown in bold and italics, respectively.

Supplementary Table S3. Primer sequences used for mutagenesis of AP-1 sites in the PAOX promoter-reporter constructs.

**Primer Name**[1]	**Nucleotide Sequence**[2]	**Nucleotide Position**[3]	**Annealing Temp. (℃)**
mAP-1-D-F	5′-ATGAACAAGCCAAGTCTTGT CAAAGCCACATGGGTAGTTG-3′	-2718~-2758	65
mAP-1-D-R	5′-TGTGGCTTTGATAAGACTTGGC TTGTTCATTTTTTTAAAATAGCT-3′	-2772~-2728	
mAP-1-P-F	5′-GCCCAGAAGACTTGC CCGACTCCCAGGCAC-3′	-1134~-1164	65
mAP-1-P-R	5′-TCGGGCAAGACTTCT GGGCGGTGGCGGG-3′	-1173~-1145	
-3126-F[3]	5′-**TCTATCGATA***GGTACC* TGAGGTCAGGTGTTCGAGACC-3′	-3127~-3105	65
-280-R[3]	5′-**CCGGAATGCC***AAGCTT* GGGGCCGGGCCGAGCCCCAC-3′	-261~-280	65

[1] mAP-1-D and mAP-1-P indicate mutations of distal and proximal AP-1 sites, respectively. F: forward primer and R: reverse primer.

[2] Sequences that were mutated are underlined.

[3] Details of these primers are presented in Supplementary Table S2.

Supplementary Table S4. Primer sequences used for PCR of ChIP and Re-IP assays.

Primer Name[1]	Nucleotide Sequence	Annealing Temp. (°C)
-2896-F	5′-CTGGATGATGGTGACAGTGG-3′	55
-2730-F[2]	5′-**TCTATCGATA***GGTACC*ATGGGTAGTTGCCACCTTG-3′	55
-1271-F[2]	5′-**TCTATCGATA***GGTACC*GTTCCCCATGGCCTGGAG-3′	65
-1100-F	5′-GGTTGGCTAGGGAGTGATGG-3′	65
-2710-R	5′-CAAGGTGGCAACTACCCATG-3′	65
-2477-R	5′-GCCTGAATCCAATAGCACGG-3′	65
-1080-R	5′-CCATCACTCCCTAGCCAACC-3′	60
-1003-R[2]	5′-**CCGGAATGCC***AAGCTT*TTTCCTTTGATTCCCTCTCG-3′	60

[1] Each primer was named based on the 5′-end nucleotide position of the PAOX promoter that annealed to each primer. The 1st nucleotide upstream of the start codon was referred to as -1. F: forward primer and R: reverse primer.

[2] Details of these primers are presented in Supplementary Table S2.

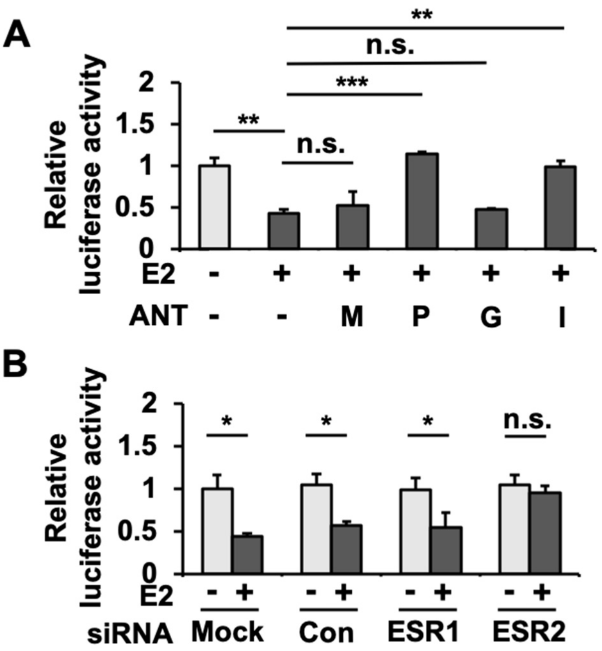

Supplementary Figure S1. Reduction in PAOX promoter activity by E2 is mediated by ESR2. MCF-7 cells were co-transfected with the pGL3-Enhancers-PAOX promoter (-3126/-280), and pRL-TK in the absence or presence of E2 and with MPP (M; 100 μM), PHTPP (P; 100 μM), G-15 (G; 100 μM), or ICI182.780 (I; 100 μM) (A), or with siRNA for Con (scrambled), ESR1, or ESR2 knockdown (B). Luciferase assays were performed as described above. Data are shown as the mean ± S.D. (n = 3), normalized to *Renilla* luciferase activity. *, $p < 0.05$; **, $p < 0.01$; and ***, $p < 0.001$ versus the PAOX promoter activity in the presence or in the absence of E2.
